# Immune Characteristic Genes and Neutrophil Immune Transformation Studies in Severe COVID-19

**DOI:** 10.3390/microorganisms12040737

**Published:** 2024-04-04

**Authors:** Zhaoming Zhou, Xin Zeng, Jing Liao, Xinfeng Dong, Yinyun Deng, Yinghui Wang, Meijuan Zhou

**Affiliations:** Department of Radiation Medicine, Guangdong Provincial Key Laboratory of Tropical Disease Research, School of Public Health, Southern Medical University, Guangzhou 510515, China; zhouzhaoming08@outlook.com (Z.Z.); zengx4463@foxmail.com (X.Z.); liaojing_smu@outlook.com (J.L.); erfeng1913@163.com (X.D.); yinyun9845@foxmail.com (Y.D.)

**Keywords:** COVID-19, SARS-CoV-2, WGCNA, neutrophil, NETs

## Abstract

As a disease causing a global pandemic, the progression of symptoms to severe disease in patients with COVID-19 often has adverse outcomes, but research on the immunopathology of COVID-19 severe disease remains limited. In this study, we used mRNA-seq data from the peripheral blood of COVID-19 patients to identify six COVID-19 severe immune characteristic genes (FPR1, FCGR2A, TLR4, S100A12, CXCL1, and L TF), and found neutrophils to be the critical immune cells in COVID-19 severe disease. Subsequently, using scRNA-seq data from bronchoalveolar lavage fluid from COVID-19 patients, neutrophil subtypes highly expressing the S100A family were found to be located at the end of cellular differentiation and tended to release neutrophil extracellular traps. Finally, it was also found that alveolar macrophages, macrophages, and monocytes with a high expression of COVID-19 severe disease immune characteristic genes may influence neutrophils through intercellular ligand–receptor pairs to promote neutrophil extracellular trap release. This study provides immune characteristic genes, critical immune pathways, and immune cells in COVID-19 severe disease, explores intracellular immune transitions of critical immune cells and pit-induced intercellular communication of immune transitions, and provides new biomarkers and potential drug targets for the treatment of patients with COVID-19 severe disease.

## 1. Introduction

Severe acute respiratory syndrome coronavirus type 2 (SARS-CoV-2)-induced coronavirus disease 2019 (COVID-19) was newly discovered at the end of 2019, and presents as a series of acute diseases, ranging from mild respiratory symptoms to severe respiratory failure, which rapidly evolved into a global pandemic by 2020 [1]. As of 4 October 2023, 771,151,224 confirmed cases of SARS-CoV-2 infections, including 6,960,783 deaths, had been reported to the World Health Organization (WHO). As a disease causing a global pandemic, studies on the innate immunopathology of COVID-19 are still limited. Severe symptoms in patients with COVID-19 are mostly characterized by respiratory failure requiring mechanical ventilation [2], which is also accompanied by multi-organ failure and systemic thrombosis [3], predisposing them to poor prognosis and even death. A number of studies have reported the presence of large numbers of neutrophils and neutrophil extracellular traps (NETs) in the blood and lungs of COVID-19 severe patients [4,5,6,7,8,9]. Neutrophil infiltration and the formation of neutrophil extracellular traps may play an important role in necrotizing inflammation in COVID-19 severe patients. NETs contribute to poor prognosis by damaging endothelial cells, stimulating exogenous and endogenous coagulation, and mediating microthrombosis and microvascular dysfunction, leading to immunothrombosis [10,11]. Therefore, it is quite important to explore the immune indicators and the immune transition from neutrophil activation to neutrophil extracellular trap formation in patients at high risk of COVID-19 severe disease to try to develop new therapeutic targets and provide new therapeutic interventions for patients with COVID-19 severe disease to reduce poor prognosis or death of patients with COVID-19 severe disease.

**Figure 1:**
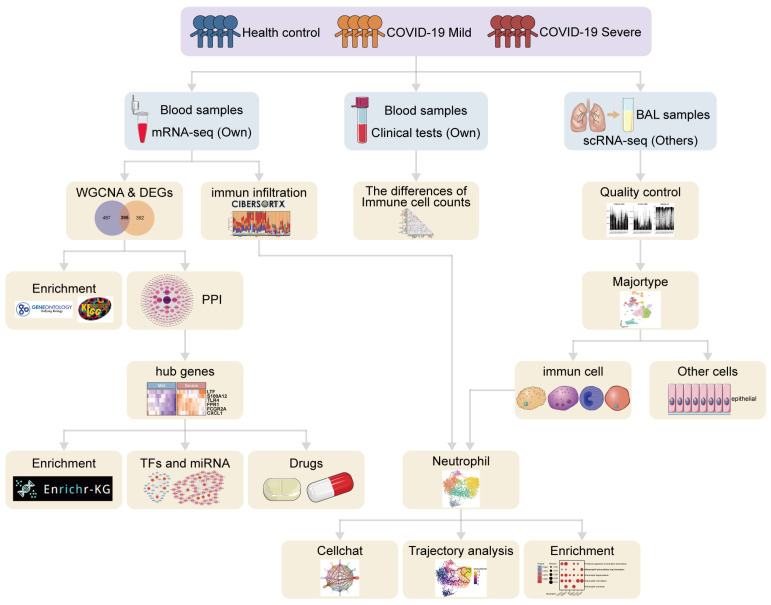
The overall analysis flow diagram of this study.

Bulk RNA sequencing (RNA-seq) and single-cell RNA sequencing (scRNA-seq) analyses provide adequate gene expression analyses to characterize COVID-19 and to explain biological pathways and critical genes not yet targeted by current therapies. Using bulk RNA-seq, Lai et al. identified M0 macrophages and neutrophils as important immune cells for COVID-19 and constructed a three-gene marker recognizing COVID-19 as a potential biomarker [12]. Wauters E et al. used bronchoalveolar lavage fluid (BALF) scRNA-seq data for detected reads mapping of the nucleocapsid protein (N)-encoding gene mainly in neutrophils and macrophages. Neutrophils might be heavily involved in viral clearance of SARS-CoV-2 [13]. Schulte-Schrepping J et al. used PBMC scRNA-seq data and found neutrophil dysfunction in patients with COVID-19, and this neutrophil dysfunction was associated with multiple potentially harmful pathways of severe COVID-19 activation [14]. In this study, we explored characteristic immune genes, immune pathways, and immune cells in COVID-19 severe patients by mRNA-seq data, along with neutrophil differentiation and the immune transition that leads to the occurrence of NETs using BALF scRNA-seq data. Finally, we also explored cellular communication between cells with a high expression of immune signature genes and neutrophils, providing new insights into the immune transition that triggers neutrophil activation to neutrophil extracellular trap formation, and providing new biomarkers and potential drug targets for COVID-19 severe patients. The analytical flow of this study is shown in Figure 1.

## 2. Materials and Methods

### 2.1. Bulk RNA Sequencing Data Collection

Bulk mRNA sequencing (mRNA-seq) data from this study contained healthy controls (n = 10), the COVID-19 severe group (n = 17), and the COVID-19 mild group (n = 14), which are available from the National Center for Biotechnology Information (NCBI) GEO database [15] under accession number GSE167930 [16]. Use the normalizeBetweenArrays function of the limma R package (version 3.52.4) [17] to normalize the data.

### 2.2. Hierarchical Cluster Analysis and Principal Component Analysis

In order to study the relationship between the severity of patients, hierarchical cluster analysis and principal component analysis were performed on all patients.

There were 41 patients in this step, including 14 patients with mild disease, 17 patients with severe disease, and 10 healthy controls. In hierarchical cluster analysis and PCA, patients with a similar expression of mRNAs tended to be close. We used the flashclust function in the WGCNA R package (version 1.72.1) [18] for hierarchical cluster analysis and the vegan R package (version 2.6.4) [19] for principal component analysis.

### 2.3. Weighted Gene Co-Expression Network Analysis

The “WGCNA” R package [18] was used to create a weighted gene co-expression network to identify critical modules and genes in the COVID-19 dataset. First, the “pickSoftThreshold” function was used to select the optimal soft threshold with scale-free topological fit index (R2) > 0.80 and good average network connectivity. The “blockwiseModules” function was used to construct the weighted gene co-expression network in one step, with a soft threshold of 12, a minModuleSize of 100, and a mergeCutHeight of 0.8. Correlations between gene modules and COVID-19 disease types were calculated using the Pearson correlation analysis method, and positively correlated modules with a *p*-value of less than 0.05 were considered to be critically associated with the phenotype and were included in subsequent analyses. Finally, the genes within the critical modules were further screened by calculating module membership (MM) and gene significance (GS) values to obtain the most relevant genes for the traits in the critical modules.

### 2.4. Identification of Differentially Expressed Genes and Critical Genes

Significant differential genes for COVID-19 severe disease in mRNA-seq data were identified using the limma R package (version 3.52.4) [17], with the criteria of a corrected *p*-value < 0.05 and |logFC| > 1.0. To screen critical genes for COVID-19 severe disease, we set up two gene clusters. Cluster 1 was the critical gene of COVID-19 severe identified by WGCNA. Cluster 2 for COVID-19 mild patients compared with COVID-19 severe patients in DEGs. Clusters 1 and 2 were intersected to find critical genes in COVID-19 severe patients.

### 2.5. Functional Enrichment Analysis

Gene Ontology (GO) (https://geneontology.org/, accessed on 28 July 2023) [20,21] is a widely used tool for annotation of gene function, containing biological pathways (BP), cellular components (CC), and molecular function (MF). KEGG (https://www.genome.jp/kegg/, accessed on 28 July 2023) [22] enrichment analysis is a practical tool for analyzing gene function and related high-level genomic functional information.

Genes were subjected to GO pathway analysis [23] using the R package org.hs.egg.db (version 3.1.0) as a background. KEGG pathway gene annotations were obtained from the Molecular Signatures Database before performing KEGG analysis. Gene enrichment results were obtained using the R package clusterProfiler (version 3.14.3) [24]. GO and KEGG pathways with *p*-values < 0.05 were considered to be significantly enriched, and the pathways were ranked according to *p*-value from smallest to largest, and the results were visualized using the ggplot2 R package (version 3.3.6) [25].

### 2.6. Protein–Protein Interaction Network Construction and Hub Gene Identification

Protein–protein interaction (PPI) network via STRING [26] (v12.0, https://cn.string-db.org/, accessed on 31 July 2023), a web-based tool for detecting protein interactions by uploading gene datasets. The critical genes obtained previously were imported into STRING and the interaction score was set to 0.4. The PPI network results were exported to Cytoscape [27] (version 3.9.1) for analysis and visualization.

The PPI network was computed using CytoHybba [28] (version 0.1), a plug-in for Cytoscape software (version 3.9.1), through five algorithms: Degree, MCC, Closeness, Betweenness, and MNC. The top 20 genes obtained from the above five algorithms were taken to be intersected, and the common genes obtained were used as hub genes. The hub genes were again KEGG, GO, and MGI Mammalian Phenotype enriched using the Enrichr-KG [29] (https://maayanlab.cloud/enrichr-kg, accessed on 1 August 2023) function of the Enrichr website (https://maayanlab.cloud/Enrichr/, accessed on 1 August 2023) to gain a greater understanding of the physiopathological functions involved in these genes.

### 2.7. Construction of TF–Gene and Gene–miRNA Interaction Networks

Identification of TF–gene and gene–miRNA interaction networks of COVID-19 severe hub genes was performed using the JASPAR database (https://jaspar2020.genereg.net/, accessed on 1 August 2023) and TarBase (version 8.0) database on NetworkAnalyst [30] (version 3.0, https://www.networkanalyst.ca, accessed on 1 August 2023). Subsequently, the network was imported into Cytoscape for visualization and analysis.

### 2.8. Identification of Target Drugs

Drug Signatures Database (DSigDB) [31] (3 August 2023), an online gene set connecting drugs and their target genes, contains 22,527 gene sets consisting of 17,389 unique compounds covering 19,531 genes. The Enrichr website provides a link to access DSigDB. In this study, the identified COVID-19 severe disease hub genes were uploaded to the Enricher website to identify drugs associated with COVID-19 severe disease.

### 2.9. Immune Infiltration Analysis

In this study, we used CIBERSORTx [32] (https://cibersortx.stanford.edu/, accessed on 30 May 2023) to identify immune cell subpopulations in mRNA-seq. CIBERSORTx is based on a linear regression model that is trained to estimate the relative abundance of individual immune cell subpopulations in a mixed cell sample from gene expression profiles of known immune cell signatures, and the algorithm can calculate 22 immune cell subpopulations. We manually merged similar cell types to generate a dataset containing 11 major cell types: B cells, dendritic cells, macrophages, mast cells, monocytes, neutrophils, natural killer cells, plasma cells, CD4+ and CD8+ T cells, and Cytotoxic T cells (Table S7). Associations between the two immune cells were compared using Pearson correlation analysis. *p* < 0.05 was considered significant.

### 2.10. Analysis of Clinical and Laboratory Test Data

Clinical and laboratory testing data for COVID-19 patients were obtained from our previous study [16]. Clinical information and laboratory test results including routine blood tests, blood gas analysis, blood chemistry, D-dimer, prothrombin time (PT), international normalized ratio (INR), and activated partial thromboplastin time (APTT) were collected. Pearson correlation analysis was used to compare the correlation between two clinical features. Independent sample *t*-test was used to compare the two groups of continuous variables. One-way ANOVA and LSD post hoc tests were used to compare three or more groups of continuous variables. *p* < 0.05 was considered significant.

### 2.11. Analysis of Single-Cell RNA Sequencing Data from Bronchoalveolar Lavage Fluid

Bronchoalveolar lavage fluid single-cell RNA sequencing (BALF scRNA-seq) data were obtained from the GSE145926 [33] dataset of the GEO database, which contains a total of 87,000 cells from three healthy controls, three COVID-19 mild patients, and six COVID-19 severe patients. Data integration, quality control, data normalization, feature selection, data scaling, linear downscaling, and UMAP clustering were performed using the standard single-cell RNA sequencing data preprocessing workflow in Seurat [34]. Cell-type annotation was performed using the marker provided with the corresponding article of the dataset (Table S8).

### 2.12. Pseudotime Analysis

CytoTRACE is a computational framework that can be used to predict cell developmental potential and differentiation status by calculating the transcriptional diversity of each cell in scRNA-seq data [35]. CytoTRACE scores were calculated for all neutrophils by applying the R package CytoTRACE (version 0.3.3). CytoTRACE scores range from 0 to 1, with higher scores indicating higher developmental potential (less differentiation) and cells in a stem cell state. The R package Monocle 3 [36] (version 1.2.9) was used to infer cell differentiation trajectories. The BALF scRNA-seq data of COVID-19 were used to construct pseudotime trajectories of neutrophil cells in healthy controls, and COVID-19 mild and severe disease. For each analysis, PCA-based downscaling was performed using differentially expressed genes for each phenotype, followed by 2D visualization using UMAP clustering. Neutrophil subtypes with high developmental potential identified by CytoTRACE were defined as pseudotime root states to characterize the differentiation trajectories and immune changes in different subtypes of neutrophils.

### 2.13. Cell-to-Cell Communication

Cell-to-cell communication analysis of multiple cell types was performed using the CellChat R package [37] (version 1.4.0). CellChat enables comparison of the number and strength of interactions between different cell populations, calculation of afferent and efferent signals for each cell population, as well as screening for critical ligand/receptor pairs between different cells. This study focuses on the signals that other cell populations afferent to neutrophils and the critical ligand/receptor pairs therein.

### 2.14. Statistical Analysis

All the data in this study were analyzed using software R (version 4.2) [38], and the data were analyzed for significance using the statistical analysis methods that come with the corresponding R package. Pearson correlation analysis was used to compare the correlation between two characteristics. Independent sample *t*-test was used to compare two groups of continuous variables. One-way ANOVA and LSD post hoc tests were used to compare three or more groups of continuous variables. Significant differences were generally considered to be significant at a *p*-value < 0.05.

## 3. Results

### 3.1. Hierarchical Cluster Analysis and Principal Component Analysis

**Figure 2:**
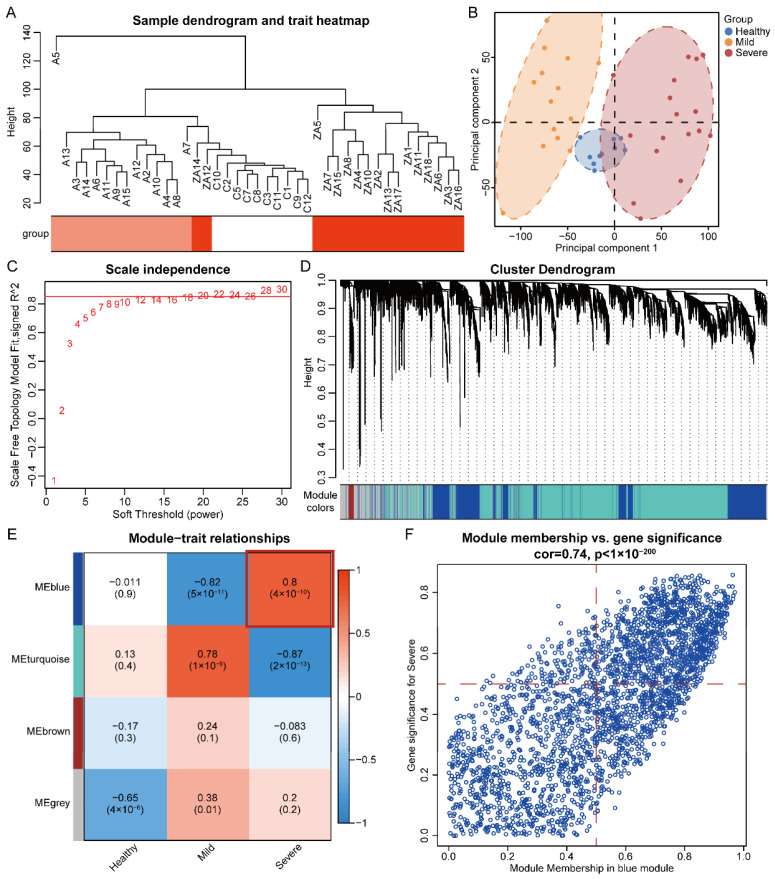
Hierarchical Clustering Analysis and principal component analysis, as well as the use of WGCNA to identify significant modules and genes of COVID-19 severe: (**A**) Hierarchical clustering of normalized bulk RNA-seq data. Red, light red and white are ZA/A/C and indicate COVID-19 severe, mild and healthy groups, respectively. These three groups can be clearly separated. (**B**) Principal component analysis (PCA) showed a clear separation of the three groups. (**C**) Soft threshold in Weighted Gene Co-Expression Network Analysis (WGCNA) of bulk RNA-seq. The red line is the soft threshold equal to 0.85. (**D**) A cluster dendrogram of module-specific colors showed 4 co-expressed gene modules, with different colors indicating that genes are grouped in different modules, each containing more than 100 genes. (**E**) Correlation heatmap between disease groupings and gene modules. (**F**) The scatter plot of Module membership vs. Gene significance in the blue co-expression module. The red dashed line shows Module membership and Gene significance equal to 0.5.

We performed unsupervised hierarchical clustering to detect similarities in gene expression profiles between severe, mild, and healthy control by the fashClust function of the WGCNA package (Figure 2A). We observed an obvious separation between those three groups, suggesting specific mRNA profile signatures for COVID-19 patients. Principal component analysis (PCA) of variant genes re-marked such separation (Figure 2B). PCA was able to distinguish severe patients and demonstrated that the severe patients were separated from the healthy control and mild patients. These two results suggest that there are differences in mRNA profiles between COVID-19 severe and mild patients.

### 3.2. Identification of Significant Modules and Genes of COVID-19 Severe by WGCNA

Next, we used the WGCNA package (version 1.7.1) to construct co-expression networks for bulk mRNA-seq normalized gene expression data. The soft threshold was set to 12 to fit a scale-free network and the maximum mean connectivity (Figure 2C). Four co-expression modules were identified, of which the blue module was positively correlated with COVID-19 severe (r = 0.8, *p* = 4 × 10^−10^) and contained 2707 genes (Figure 2D,E). We calculated the expression correlation of module feature vectors with genes to obtain module membership (MM) and gene significance (GS). Based on the cut-off criteria MM > 0.5 and GS > 0.5, 882 genes with high connectivity in the blue module were identified (Figure 2F).

### 3.3. Identification and Enrichment of Critical Genes for COVID-19 Severe Disease

**Figure 3:**
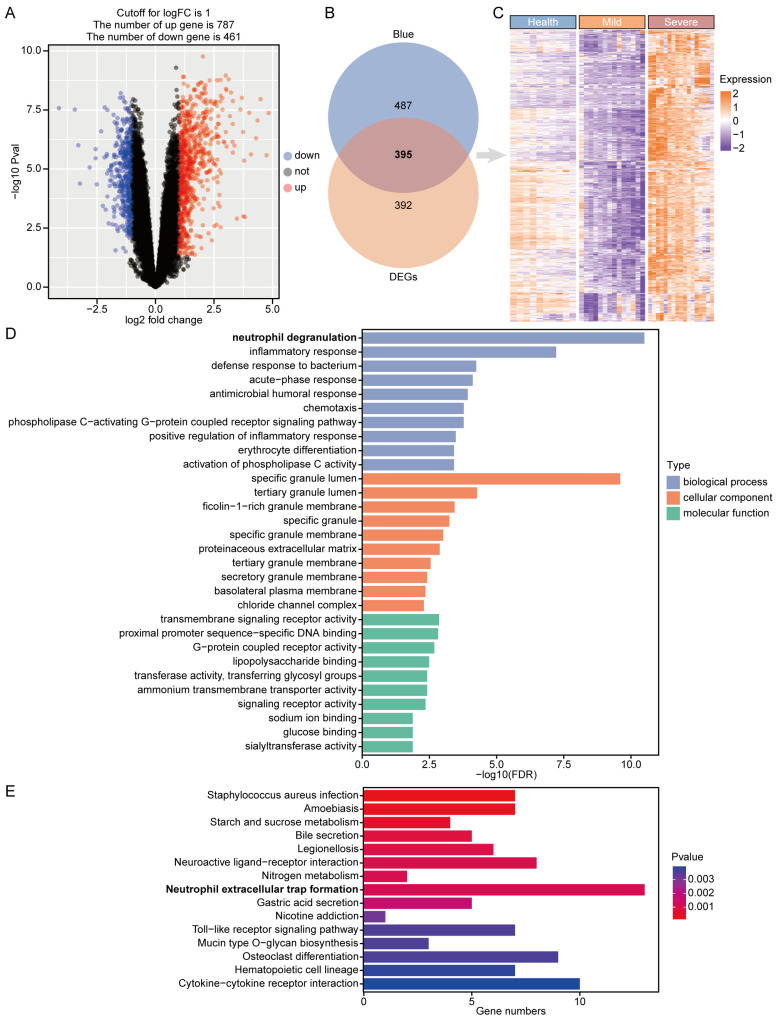
Differential gene expression analysis, identification, and enrichment of critical genes in COVID-19 severe: (**A**) Volcano plot of differentially expressed genes (DEGs) between COVID-19 severe and mild. (**B**) The 787 DEGs of COVID-19 severe disease and the 882 high connectivity genes of the blue module were taken to be intersected, and 395 critical genes were obtained. (**C**) Heatmap of 395 critical genes expression in different disease types. (**D**) GO enrichment analysis of 395 critical genes in COVID-19 severe. (**E**) KEGG enrichment analysis of 395 critical genes in COVID-19 severe. Bolded font emphasizes neutrophil-associated biological processes and pathways.

This study focused on COVID-19 severe disease; therefore, the differentially expressed genes were calculated by comparing the expressed genes of COVID-19 severe and mild disease. Under Padj < 0.05 and |logFC| > 1 screening criteria, 1248 DEGs were obtained, including 787 up-regulated and 395 down-regulated genes (Table S1, Figure 3A). Table 1 demonstrates the top 10 differentially expressed genes that were up-regulated.

Intersecting the up-regulated genes (787 differentially expressed genes in COVID-19 severe) with the 882 highly connected genes in the blue module identified by WGCNA as being positively correlated with COVID-19 severe resulted in 395 critical genes (Table S2, Figure 3B). A heatmap showing the expression of these 395 critical genes in different disease types is seen in Figure 3C.

GO and KEGG enrichment analysis was performed on 395 critical genes of COVID-19 severe to understand their biological pathways (Tables S3 and S4). The top ten GO terms for biological process, cellular component, and molecular function are shown in Figure 3D. The biological processes of GO are mainly enriched in neutrophil degranulation, inflammatory response, defense response to bacterium, acute-phase response, and antimicrobial humoral response, which are all acute inflammation-related pathways. The cellular components of GO are mainly enriched in neutrophil granules, such as specific granule lumen, tertiary granule lumen, ficolin-1-rich granule membrane, specific granule, and specific granule membrane. The molecular functions of GO are mainly enriched in transmembrane signaling receptor activity, proximal promoter sequence-specific DNA binding, G-protein coupled receptor activity, lipopolysaccharide binding, and transferase activity, transferring glycosyl groups. Figure 3E shows the top 15 KEGG-enriched pathways. KEGG analysis found that critical genes were mainly enriched in Staphylococcus aureus infection, amoebiasis, starch and sucrose metabolism, bile secretion, legionellosis, nicotine addiction, neuroactive ligand–receptor interaction, nitrogen metabolism, and neutrophil extracellular trap formation. Both GO and KEGG were enriched to neutrophil-associated pathways, suggesting that neutrophils may play an important role in COVID-19 severe.

### 3.4. Construction of PPI Network and Identification of Hub Gene

**Figure 4:**
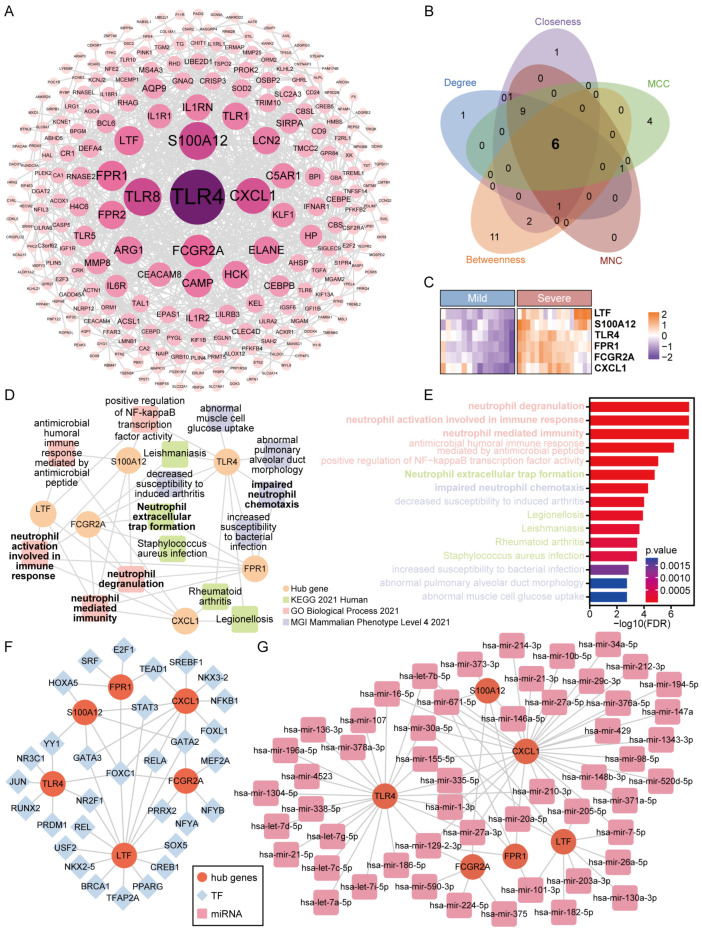
Identifying the key genes for COVID-19 and identifying their associated TFs and miRNA: (**A**) Protein–protein interaction network was created using STRING and visualized in Cytoscape. The larger the degree, the larger the node, the darker the color, and the more centered the position. (**B**) Cytohubba plugin further screens for hub genes in the network. The Venn diagram shows the number of hub genes and the algorithm used. (**C**) Heatmap of hub genes expression in COVID-19 mild and severe. (**D**,**E**) GO, KEGG, and MGI enrichment results for hub genes. (**F**) TF–gene interaction network. Herein, the circle nodes are genes (red); the diamond nodes are TFs (Blue). (**G**) Gene–miRNA interaction network. Herein, the circle nodes are genes (red); the square nodes are miRNA (pink).

Protein–protein interaction networks (251 nodes and 751 edges) for these critical genes were constructed using STRING (v12.0; https://cn.string-db.org, accessed on 31 July 2023). Thereafter, the network was imported into the Cytoscape version 3.9.1 for analysis and visualization (Figure 4A). Based on the Degree, MCC, Closeness, Betweenness, and MNC algorithms of the CytoHubba plug-in, six genes (FPR1, FCGR2A, TLR4, S100A12, CXCL1, and LTF) were confirmed as hub genes related to COVID-19 severe (Figure 4B, Table S5). All topological features of hub genes are shown in Table 2. A heatmap shows the expression of six hub genes in mild and severe of COVID-19 (Figure 4C). These six hub genes may be the characteristic immune genes of COVID-19 severe, which can be used as potential biomarkers for the diagnosis and treatment of COVID-19 severe.

Hub genes were enriched using the online enrichment tool Enrichr-KG, which is enriched in neutrophil degranulation, neutrophil activation involved in immune response, neutrophil-mediated immunity, antimicrobial humoral immune response mediated by antimicrobial peptide, positive regulation of NF-kappaB transcription factor activity, neutrophil extracellular trap formation, impaired neutrophil chemotaxis, decreased susceptibility to induced arthritis, legionellosis, leishmaniasis, rheumatoid arthritis, Staphylococcus aureus infection, increased susceptibility to bacterial infection, abnormal pulmonary alveolar duct morphology, and abnormal muscle cell glucose uptake (Figure 4D,E). The enrichment results contained five neutrophil-related pathways, suggesting that the immunopathological effects of hub genes are closely related to neutrophils; it further suggests that neutrophils may play an important role in COVID-19 severe.

### 3.5. Construction of TF–Gene and Gene–miRNA Interaction Networks and Drug Identification

NetworkAnalyst was used to identify the TF–gene and gene–miRNA interaction networks for hub genes. The TF–gene network consists of 31 TF genes and 6 hub genes (Figure 4F). LTF was regulated by 15 TF genes and CXCL1 was regulated by 10 TF genes. In addition, we found that FOXC1 had high connectivity in the TF–gene interaction network, regulating six hub genes simultaneously. The gene–miRNA network has 59 nodes and 78 edges, where 6 of the nodes are hub genes and 53 are miRNAs (Figure 4G). Among these miRNAs, hsa-mir-129-2-3p was connected with CXCL1, TLR4, FPR1, and FCGR2A; hsa-mir-20a-5p and hsa-mir-671-5p were associated with CXCL1, LTF, S100A12, and FPR1; and hsa-mir-27a-3p was connected with CXCL1, TLR4, S100A12, and FCGR2A. These four miRNAs are also recognized as the critical miRNAs.

Based on hub genes, a total of 409 drug compounds were identified through the DSigDB database on the Enrichr website, which identifies drugs associated with severe illness in COVID-19 (Table S6). The top ten drug compounds were screened according to the *p*-value (Table 2).

**Table 2:** The top ten gene-targeted drugs in the COVID-19 severe.

Term	*p*-Value	Combined Score	Genes
trimethoprim BOSS	1.68 × 10^−5^	1158.315235	CXCL1; TLR4; LTF
Muramyl Dipeptide CTD 00005307	1.73 × 10^−5^	5475.88964	CXCL1; TLR4
Adenylyl sulfate BOSS	2.06 × 10^−5^	4896.915145	TLR4; LTF
6-Deoxy-D-galactose BOSS	3.94 × 10^−5^	3265.179181	TLR4; LTF
N-Formyl-Met-Leu-Phe BOSS	6.42 × 10^−5^	2407.70308	FPR1; TLR4
Lysergide BOSS	7.06 × 10^−5^	2270.521513	TLR4; LTF
methacholine BOSS	9.13 × 10^−5^	1932.575689	CXCL1; TLR4
SODIUM SULFATE BOSS	1.55 × 10^−4^	1387.901255	CXCL1; TLR4
Heparitin BOSS	2.97 × 10^−4^	918.6400252	CXCL1; TLR4
Hydroxyzine dihydrochloride BOSS	3.66 × 10^−4^	803.1179907	TLR4; LTF

### 3.6. Immune Infiltration for Diverse Disease Severity in COVID-19

**Figure 5:**
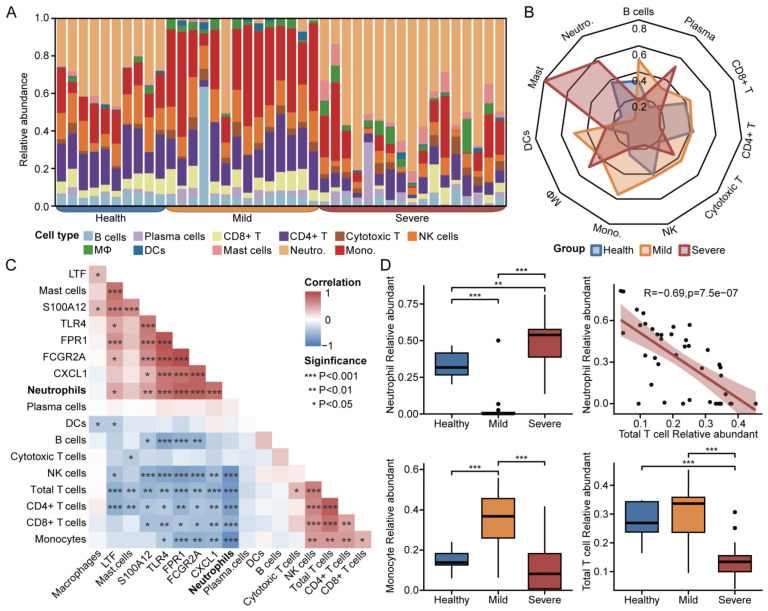
Immune infiltration for diverse disease severity in COVID-19: (**A**) Relative abundance of a variety of immune cells. (**B**) The normalized average abundance for patients with diverse disease severity based on RNA-seq. (**C**) Heatmap of correlation between hub genes and immune cells. (**D**) Box plots and correlation scatter plots of neutrophils, monocytes, and total T cells. (* *p* < 0.05, ** *p* < 0.01, *** *p* < 0.001.).

We used the online database CIBERSORTx for immune cell infiltration analysis of bulk mRNA-seq data. Figure 5A demonstrates the relative abundance of various immune cells, with a significant increase in neutrophils and a significant decrease in monocytes, CD4+ T, and CD8+ T cells in severe patients compared with mild patients (Figure 5B,D). A comparison of the association between each hub gene and immune cells using Pearson correlation analysis showed that the hub genes were all positively correlated with neutrophils and negatively correlated with both CD4+ T and total T cells (Figure 5C). In addition, monocytes, NK cells, CD4+ T cells, CD8+ T cells, and total T cells were significantly negatively correlated with neutrophils (Figure 5C,D). Combined with the previous enrichment results, it suggests that neutrophils play a non-negligible role in COVID-19 severe, and that targeting neutrophils for the treatment of patients with COVID-19 severe may have promising results.

### 3.7. Clinical Metrics and Laboratory Test Results in Patients with COVID-19

**Figure 6:**
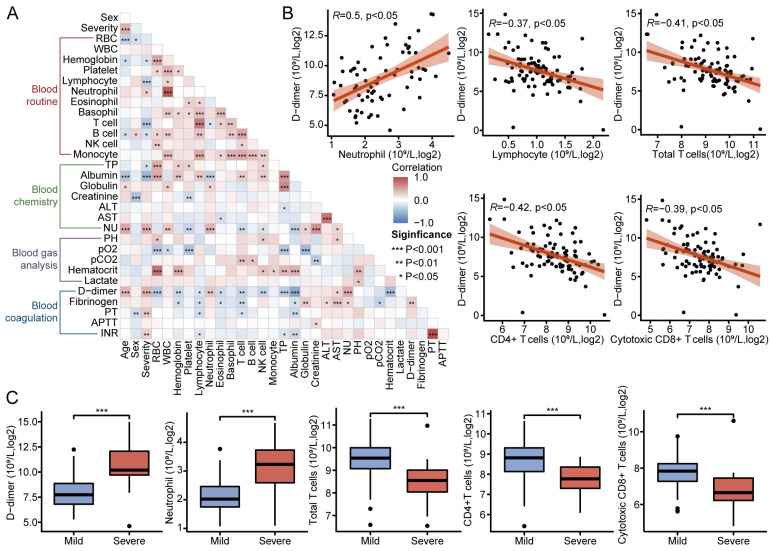
Clinical metrics and laboratory test results in patients with COVID-19: (**A**) Heatmap of correlation between clinical and laboratory test results. (**B**) Scatter plot of D-dimer correlation with neutrophils, lymphocytes, total T cells, CD4+ T cells, and Cytotoxic CD8+ T cells. (**C**) Box plots of D-dimer, neutrophil, total T cells, CD4+ T cells, and Cytotoxic CD8+ T cells in COVID-19 mild and severe. (* *p* < 0.05, ** *p* < 0.01, *** *p* < 0.001.).

To better understand the immune characteristics of COVID-19 severe patients, we analyzed the clinical and laboratory test results of the COVID-19 patients. The heatmap demonstrates the correlation between clinical tests and laboratory test results (Figure 6A). Three of the five indicators of the coagulation routine were positively correlated with severity, suggesting a risk of endogenous coagulation in COVID-19 severe patients. Interestingly, the concentration of D-dimer, a critical indicator of coagulation in COVID-19 patients, was positively correlated with neutrophil counts and negatively correlated with lymphocyte, total T, CD4+ T, and cytotoxic CD8+ T cell counts (Figure 6B). Figure 6C shows that COVID-19 severe patients had significantly higher D-dimer concentrations, neutrophil counts, and significantly lower total T, CD4+ T, and cytotoxic CD8+ T cell counts compared to COVID-19 mild patients. The changes in immune cells were consistent with the results of the immune infiltration analysis of bulk mRNA-seq data.

### 3.8. BALF Single-Cell Sequencing Reveals Internal Immune Shifts in Neutrophils from COVID-19 Patients

**Figure 7:**
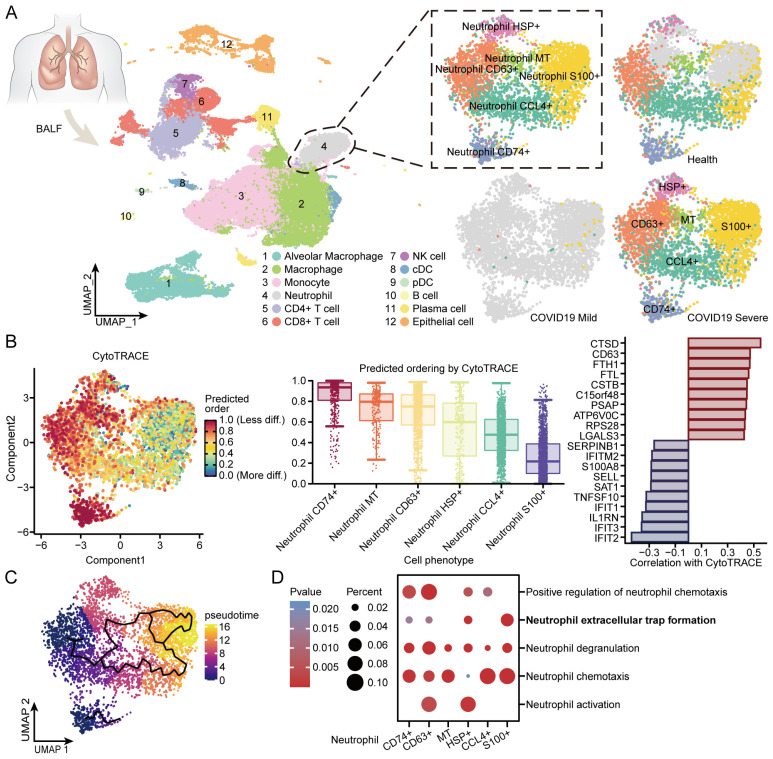
Distribution of neutrophil subgroup and immune transformation in BALF scRNA-seq: (**A**) An overview of UMAP clusters of diverse immune cells and particularly the subgroup of neutrophils in health controls and COVID-19 patents. A considerably large number of neutrophils was observed in COVID-19 severe patients. cDC, conventional dendritic cell. pDC, plasmacytoid dendritic cell. (**B**) UMAP plots depicting the distribution of CytoTRACE scores among subgroup of neutrophils (left). Dark green indicates lower scores (low stemness) while dark red indicates higher scores (high stemness). CytoTRACE predicted the cell differentiation potential of subgroup of neutrophils (Middle). Genes correlated with more differentiated and less differentiated cells predicted by CytoTRACE (Right). (**C**) Pseudotime of neutrophil subgroup. (**D**) Enrichment results of neutrophil-associated pathways in neutrophil subgroup.

To investigate the role of neutrophils in COVID-19 patients, we obtained single-cell sequencing data of bronchoalveolar lavage fluid (BALF scRNA-seq) from the GSE145926 dataset in the GEO database for three healthy controls, three COVID-19 mild cases, and six COVID-19 severe cases. Using these data, we identified 12 major cell types, extracted their neutrophil, and further identified six subsets of neutrophils (Figure 7A and Figure S1). The distribution of neutrophils across disease states was consistent with the results of bulk mRNA-seq data on immune infiltration, with Neutrophil CD63+ and Neutrophil S100+ being increased in COVID-19 severe compared to healthy controls. The differentiation potential of the six neutrophil subgroups was estimated using CytoTRACE (version 0.3.3), and the three neutrophil subtypes, Neutrophil CD74+, Neutrophil MT, and Neutrophil CD63+, had high differentiation potential and were in the early stage of differentiation, while Neutrophil HSP+, Neutrophil CCL4+, and Neutrophil S100+ had low differentiation potential and were in the late stage of differentiation (Figure 7B). Analysis of neutrophils using the R package Monocle 3 (version 1.2.9) further demonstrated the differentiation trends of these six neutrophils (Figure 7C). We performed an enrichment analysis of differentially expressed genes in each subset of neutrophils to see how these genes are enriched in neutrophil-related pathways (Table S9). We found that Neutrophil S100+ at a late stage of differentiation was significantly enriched for neutrophil extracellular trap formation (Figure 7D). These results suggest that there may be a gradual intracellular process of gradual change in neutrophils from activation to the occurrence of extracellular trap formation in COVID-19 severe disease, and that the inhibition of this process of change to minimize the occurrence of NETs may be a potential therapeutic approach to improve the symptoms and prognosis of COVID-19 severe patients.

### 3.9. Identification of the Distribution and Cellular Communication of Hub Genes in Immune Cells from Patients with COVID-19

**Figure 8:**
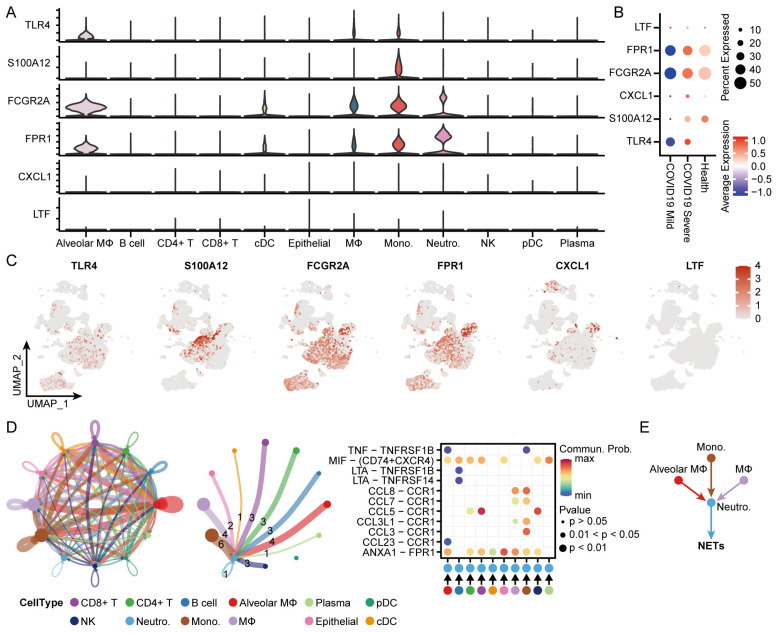
Single-cell sequencing explores the expression of hub genes and cellular communication in immune cells: (**A**,**C**) Expression of hub genes in immune cells in BALF scRNA-seq. (**B**) Average expression of hub genes in different disease groups in BALF scRNA-seq. (**D**) Cell–cell communications analysis and vital ligand–receptor interactions in immune cells, with numbers on the lines representing the number of interactions. (**E**) Diagram of the cell–cell interactions of Monocytes, Alveolar macrophages, Macrophages, and Neutrophils.

We used BALF scRNA-seq data to assess the specific expression of hub genes in different immune cells and in different disease types. TLR4 was expressed in alveolar macrophages, macrophages, and monocytes; S100A12 was highly expressed in macrophages; FCGR2A and FPR1 were highly expressed in alveolar macrophages, cDCs, macrophages, and monocytes versus neutrophils; CXCL1 was expressed in epithelial cells and neutrophils; and LTFs were not significantly expressed (Figure 8A,C). In addition, consistent with that of bulk mRNA-seq, hub genes in BALF scRNA-seq had the highest average expression in COVID-19 severe patients (Figure 8B).

Using the CellChat package to explore the cellular communication network between neutrophils and other immune cells, we found that alveolar macrophages, macrophages, and monocytes with generally high expressions of hub genes had more cell-to-cell communication toward neutrophils (Figure 8D). Combined with the results of neutrophil differentiation and enrichment analyses, we hypothesized that alveolar macrophages, macrophages, and monocytes may be present in the immunopathology of COVID-19 severe, influencing neutrophils through intercellular ligand–receptor pairs to promote the occurrence of extracellular trapping of neutrophils (Figure 8E).

## 4. Discussion

COVID-19 is a highly infectious disease that has spread to many countries and regions around the globe and has affected the social and economic well-being of many countries and regions [39,40]. Severe SARS-CoV-2 infection induces an intense inflammatory response that triggers the recruitment and infiltration of neutrophils in different organs and the formation of neutrophil extracellular traps (NETs), which leads to a variety of serious consequences of SARS-CoV-2 infection, such as acute respiratory failure (ALI), acute respiratory distress (ARDS), cytokine storm (CS), pulmonary thrombosis, and multiple organ damage (MOD) [41]. Therefore, it is important to explore the immune characteristic genes of COVID-19 severe and the immune transformation of neutrophils for the treatment of COVID-19 severe, reducing death and improving prognosis. We performed a study using peripheral blood mRNA-seq data from 41 COVID-19 patients to obtain the immune-characterized genes of COVID-19 severe disease, namely, the hub genes (FPR1, FCGR2A, TLR4, S100A12, CXCL1, and LTF).

FPR1 is a pattern recognition receptor, mainly expressed in myeloid cells, which mediates phagocytic responses to microbial invasion of the host and plays an important role in host defense and inflammatory responses [42]. FPR1 was found to induce blood neutrophil activation in COVID-19 patients, triggering neutrophil-mediated inflammation and end-organ damage [43]. It has also been found that T cells and myeloid cells from COVID-19 patients may trigger an inflammatory storm in the lungs through intercellular communication via the ANXA1/FPR1 receptor–ligand signaling pathway [44] The study by Lee H et al. [45] also concluded that FPR1 is a suitable target for appropriate assessment of COVID-19 severity and provision of therapeutic agents. FCGR2A is a member of the family of genes encoding immunoglobulin Fc receptors present on the surface of many immune-responsive cells. The protein it encodes is a cell surface receptor for phagocytic cells such as macrophages and neutrophils, and is involved in the phagocytosis and clearance of immune complexes [46]. Thromboembolic events are an important cause of death in critically ill patients with COVID-19, and platelet-mediated immune thrombosis within the plasma of COVID-19 patients can be reversed by blocking FCGR2A-Syk pathway signaling on platelets [47]. It has also been found that immune thrombocytopenia was induced after vaccination with the COVID-19 vaccine Vaxzevria, and that the resulting antibodies directed against platelet factor-4 (PF4)/heparin complex induced platelet activation by binding to the FCGR2A receptor on the surface of platelets, triggering thromboembolism and even death [48]. The protein encoded by TLR4 is a member of the Toll-like receptor (TLR) family, which acts as a type I transmembrane protein that recognizes lipopolysaccharides and initiates intracellular signaling through the NF-κB or JNK/SAPK signaling pathways, playing an important role in pathogen recognition and activation of innate immunity [49]. It was found that the SARS-CoV-2 spike protein may trigger an inflammatory response through TLR4 signaling [50]. Obese patients with severe COVID-19 induced inflammation through saturated fatty acid activation of the TLR4 pathway, and additionally pro-inflammatory cytokines may promote hypertensive responses in the hypothalamus by triggering activation of the TLR4/MyD88/NF-ĸB pathway; therefore, activation of the TLR4 pathway in COVID-19 patients may be associated with the poor prognosis of patients, and inhibition of the TLR4/NF-ĸB pathway reduces the immune response to pathogen-associated molecular patterns (PAMPs) and danger-associated molecular patterns (DAMPs) in COVID-19 patients, thereby improving the condition and prognosis of COVID-19 patients [51,52]. The protein encoded by S100A12 is a member of the S100 family of proteins containing 2 EF-hand calcium-binding motifs. S100 proteins are localized in the cytoplasm and nucleus of a wide range of cells and are involved in the regulation of various cellular processes such as cell cycle progression and differentiation. S100A12 is highly abundant in neutrophils during acute inflammation and is associated with immune regulation [53]. The study by Lei et al. found a strong correlation between S100A12 expression and indicators of disease severity and prognosis in COVID-19 patients, which is a valuable marker of COVID-19 severity and assessment of prognosis [54]. S100A12, the gene encoding EN-RAGE, was analyzed in COVID-19 proteomic analysis of lung tissue from patients who had died, and it revealed a positive correlation between EN-RAGE abundance and inflammation severity [55]. EN-RAGE was also found to be a marker of inflammation in severe sepsis in the analysis of scRNA-seq data, and S100A12 was highly expressed by classical monocytes and myeloid cells from COVID-19 severe patients [56]. CXCL1 is a member of the CXC subfamily of chemokines. The encoded protein is a cytokine that signals through the G protein-coupled receptor CXCR2. This protein plays a role in inflammation and acts as a chemoattractant for neutrophils, causing migration and infiltration of neutrophils to sites of high expression. CXCL1 is also one of the components of the SARS-CoV-2-induced cytokine storm and is elevated in the blood of patients with severe COVID-19, mobilizing bone marrow neutrophils, resulting in increased blood neutrophilia and recruiting to the lungs, releasing neutrophils outside the neutrophil packet traps, which causes damage to the respiratory and circulatory systems [57,58]. LTF (Lactoferrin) is a member of the transferrin gene family and its protein product is found in the secondary granules of neutrophils. This protein is the major iron-binding protein in milk and human secretions, has antimicrobial activity, and is an important component of the non-specific immune system. The protein has a wide range of properties including regulation of iron homeostasis, host defense against a wide range of microbial infections, anti-inflammatory activity, regulation of cell growth and differentiation, and prevention of cancer development and metastasis. The protein and its peptides have been found to possess antibacterial, antiviral, antifungal, and antiparasitic activities [59]. Lactoferrin is currently receiving widespread attention as an antiviral agent for the treatment of new crowns; it works by inhibiting viral entry into cells at the source through the inhibition of heparan sulfate proteoglycans (HSPGs), which promotes viral cell attachment [60]. LTF acts as an antiviral substance and is produced after coronavirus contact with airway epithelial cells and produces cytokines and chemokines that recruit inflammatory cells and influence adaptive immunity [61].

Gene pathway enrichment analysis of hub genes. GO biological processes are enriched in neutrophil-associated pathways such as neutrophil degranulation, and neutrophil activation involved in immune response and neutrophil-mediated immunity. KEGG is enriched in inflammatory response pathways triggered by infections such as neutrophil extracellular trap formation, legionellosis, and leishmaniasis. The immune signature genes of COVID-19 severe disease are highly enriched in the neutrophil-associated pathway, suggesting that neutrophils may be involved in the progression of COVID-19 patients. The immune system defends against invading pathogens, but it can also trigger a severe inflammatory response. Analysis of immune infiltration by mRNA-seq revealed that neutrophils were highly infiltrated in COVID-19 severe disease and that the expression of COVID-19 severe disease immune signature genes was positively correlated with the proportion of neutrophil infiltration. Clinically detected neutrophil counts in COVID-19 patients were consistent with the results of mRNA-seq immune infiltration analysis. This further emphasizes the important role of immune-characterized genes and neutrophils in regulating the immune microenvironment in COVID-19 severe patients. It has been demonstrated that circulating and lung neutrophil counts and activation correlate with the severity of COVID-19 [62] and that the neutrophil-to-lymphocyte ratio (NLR) is an important predictor of disease severity in patients with COVID-19 [63]. Neutrophil extracellular traps (NETs) were demonstrated in the blood of many COVID-19 hospitalized patients [64]. Histopathologic studies also revealed the presence of neutrophil infiltration in the lungs of patients who died of COVID-19 [5]. Neutrophil infiltration and formation of neutrophil extracellular traps may play an important role in necrotizing inflammation in COVID-19 severe patients, where NETs mediate microthrombosis and microvascular dysfunction by damaging endothelial cells and stimulating exogenous and endogenous coagulation [10,11]. Therefore, neutrophils not only have predictive value, but also moderate inhibition of their proliferative infiltration, and NETs are potentially important therapeutic strategies to alleviate COVID-19 [65,66].

Finally, to further understand the role of neutrophils in COVID-19 severe disease, we used scRNA-seq data to identify neutrophil subtypes, and explored the immune transformation of neutrophils and the intercellular communication between immune cells with a high expression of immune-characterized genes and neutrophils. Our finding that the percentage of neutrophil numbers in BALF scRNA-seq for different disease levels is consistent with the results of peripheral blood mRNA-seq immune infiltration further emphasizes the potential role of neutrophils in COVID-19 severe disease. The six neutrophil subtypes we identified have distinct-role phenotypes and the phenotypes correspond to the order of differentiation. The neutrophil subtypes characterized by the high expression of the S100 family at the end of differentiation have a pro-inflammatory phenotype that is enriched in neutrophil extracellular trap formation pathways, and it is suggested that there is a series of immune-related changes in COVID-19 patients who have a large number of neutrophils, with NETs being ultimately released. NET formation is a key link between inflammation and thrombosis [67], and defective NET degradation in COVID-19 severe patients further promotes the accumulation of NETs [68], which leads to immune thrombosis after adhesion to platelets [69]. During COVID-19 virus infection, the sustained activation of monocytes, macrophages, and neutrophils is closely associated with excessive release of NETs [68] Monocytes or macrophages may be the trigger for inducing immunothrombosis in COVID-19 patients [70]. Our study also found that monocytes, macrophages, and alveolar macrophages with a high expression of COVID-19 severe disease immune-characterized genes may be involved in the immune transformation of neutrophils through intercellular ligand-receptor pairs.

Overall, we identified immune-characterized genes and key immune cells in COVID-19 severe disease by bulk RNA-seq, explored the immune transition of key immune cells using single-cell RNA-seq, and identified ligand–receptor pairs for cell-to-cell communication between immune cells with a high expression of immune characterized genes and key immune cells. These results provide new insights into the immune transformation of neutrophils in COVID-19 patients and offer new biomarkers and potential drug targets for the treatment and improved prognosis of COVID-19 severe patients. These new biomarkers and drug targets may also provide some hints for future studies of other COVID-19 virus variants to further minimize the impact of the COVID-19 pandemic on human health.

## 5. Conclusions

In this study, we focused on the differences between COVID-19 severe and mild disease, and identified six hub genes (FPR1, FCGR2A, TLR4, S100A12, CXCL1, and LTF) for COVID-19 severe disease based on mRNA-seq data, explored the immune pathways involved in COVID-19 severe disease, and found that neutrophils and neutrophil extracellular traps (NETs) may play important roles in COVID-19 severe disease. BALF scRNA-seq data were utilized to explore the internal immune transition of neutrophils to NETs, and the cellular communication between cells with high hub gene expression and neutrophils. These studies provide new insights into the immune transition that triggers neutrophil activation to neutrophil extracellular trap formation, providing new biomarkers and potential drug targets for treatment and improved prognosis in patients with COVID-19 severe disease.

## 6. Limitations

This study also has many limitations. Firstly, although multiple data were used in this study, the sample size used was limited and could not cover all COVID-19 patients, and there may be individual differences. Secondly, the immune characteristic genes and drugs screened in this study were predicted using bioinformatics analysis, and their specific effects need to be verified experimentally. Finally, the immunological transformation of neutrophils and the specific mechanism of other immune cells involved in the immunological transformation of COVID-19 severe neutrophils need to be further investigated and verified by in vivo experiments.

## Figures and Tables

**Table 1:** The top ten differentially expressed genes (DEGs) in COVID-19 severe.

Gene	logFC	Pval	FDR	Regulate
EGLN1	2.02491	1.72 × 10^−10^	2.12 × 10^−6^	UP
MANSC1	3.00036	1.10 × 10^−9^	2.31 × 10^−6^	UP
PDK3	1.18087	1.24 × 10^−9^	2.31 × 10^−6^	UP
HIST2H2BE	1.21761	1.26 × 10^−9^	2.31 × 10^−6^	UP
KCNJ2	3.12549	1.61 × 10^−9^	2.31 × 10^−6^	UP
TGFA	2.78342	1.71 × 10^−9^	2.31 × 10^−6^	UP
SULT1B1	2.6309	1.75 × 10^−9^	2.31 × 10^−6^	UP
CMTM1	2.31296	1.77 × 10^−9^	2.31 × 10^−6^	UP
PPP1R3D	1.93043	2.15 × 10^−9^	2.31 × 10^−6^	UP
KBTBD7	1.96831	2.20 × 10^−9^	2.31 × 10^−6^	UP

## Data Availability

The datasets GSE167930 and GSE145926 presented in this study can be found in NCBI Gene Expression Omnibus.

## Supplementary Materials

The following supporting information can be downloaded at: https://www.mdpi.com/article/10.3390/microorganisms12040737/s1, Figure S1: BAL scRNA-seq cell-type definition and disease-type distribution; Table S1: DEGs associated with COVID-19 in GSE167930; Table S2: The intersection of DEGs and blue module genes in GSE167930; Table S3: GO pathways analysis results; Table S4: KEGG pathways analysis results; Table S5: The top 20 genes in the PPI network were calculated using five algorithms; Table S6: COVID-19 severe gene-targeted drugs; Table S7: Relative abundance of major immune cell types; Table S8: List of marker genes for cell types; Table S9: GO and KEGG pathways in neutrophil subtypes.

## References

[1] Dong E., Du H., Gardner L. (2020). An Interactive Web-Based Dashboard to Track COVID-19 in Real Time. Lancet Infect. Dis..

[2] Marini J.J., Gattinoni L. (2020). Management of COVID-19 Respiratory Distress. JAMA.

[3] Zhou F., Yu T., Du R., Fan G., Liu Y., Liu Z., Xiang J., Wang Y., Song B., Gu X. (2020). Clinical Course and Risk Factors for Mortality of Adult Inpatients with COVID-19 in Wuhan, China: A Retrospective Cohort Study. Lancet.

[4] Guan J., Wei X., Qin S., Liu X., Jiang Y., Chen Y., Chen Y., Lu H., Qian J., Wang Z. (2020). Continuous Tracking of COVID-19 Patients’ Immune Status. Int. Immunopharmacol..

[5] Varga Z., Flammer A.J., Steiger P., Haberecker M., Andermatt R., Zinkernagel A.S., Mehra M.R., Schuepbach R.A., Ruschitzka F., Moch H. (2020). Endothelial Cell Infection and Endotheliitis in COVID-19. Lancet.

[6] Liu J., Liu Y., Xiang P., Pu L., Xiong H., Li C., Zhang M., Tan J., Xu Y., Song R. (2020). Neutrophil-to-Lymphocyte Ratio Predicts Critical Illness Patients with 2019 Coronavirus Disease in the Early Stage. J. Transl. Med..

[7] Buja L.M., Wolf D.A., Zhao B., Akkanti B., McDonald M., Lelenwa L., Reilly N., Ottaviani G., Elghetany M.T., Trujillo D.O. (2020). The Emerging Spectrum of Cardiopulmonary Pathology of the Coronavirus Disease 2019 (COVID-19): Report of 3 Autopsies from Houston, Texas, and Review of Autopsy Findings from Other United States Cities. Cardiovasc. Pathol..

[8] Middleton E.A., He X.-Y., Denorme F., Campbell R.A., Ng D., Salvatore S.P., Mostyka M., Baxter-Stoltzfus A., Borczuk A.C., Loda M. (2020). Neutrophil Extracellular Traps Contribute to Immunothrombosis in COVID-19 Acute Respiratory Distress Syndrome. Blood.

[9] Leppkes M., Knopf J., Naschberger E., Lindemann A., Singh J., Herrmann I., Stürzl M., Staats L., Mahajan A., Schauer C. (2020). Vascular Occlusion by Neutrophil Extracellular Traps in COVID-19. EBioMedicine.

[10] Dou Q., Wei X., Zhou K., Yang S., Jia P. (2020). Cardiovascular Manifestations and Mechanisms in Patients with COVID-19. Trends Endocrinol. Metab..

[11] Tomar B., Anders H.-J., Desai J., Mulay S.R. (2020). Neutrophils and Neutrophil Extracellular Traps Drive Necroinflammation in COVID-19. Cells.

[12] Lai G., Liu H., Deng J., Li K., Xie B. (2022). A Novel 3-Gene Signature for Identifying COVID-19 Patients Based on Bioinformatics and Machine Learning. Genes.

[13] Wauters E., Van Mol P., Garg A.D., Jansen S., Van Herck Y., Vanderbeke L., Bassez A., Boeckx B., Malengier-Devlies B., Timmerman A. (2021). Discriminating Mild from Critical COVID-19 by Innate and Adaptive Immune Single-Cell Profiling of Bronchoalveolar Lavages. Cell Res..

[14] Schulte-Schrepping J., Reusch N., Paclik D., Baßler K., Schlickeiser S., Zhang B., Krämer B., Krammer T., Brumhard S., Bonaguro L. (2020). Severe COVID-19 Is Marked by a Dysregulated Myeloid Cell Compartment. Cell.

[15] Edgar R., Domrachev M., Lash A.E. (2002). Gene Expression Omnibus: NCBI Gene Expression and Hybridization Array Data Repository. Nucleic Acids Res..

[16] Zhou Z., Zhou X., Cheng L., Wen L., An T., Gao H., Deng H., Yan Q., Zhang X., Li Y. (2021). Machine Learning Algorithms Utilizing Blood Parameters Enable Early Detection of Immunethrombotic Dysregulation in COVID-19. Clin. Transl. Med..

[17] Ritchie M.E., Phipson B., Wu D., Hu Y., Law C.W., Shi W., Smyth G.K. (2015). Limma Powers Differential Expression Analyses for RNA-Sequencing and Microarray Studies. Nucleic Acids Res..

[18] Langfelder P., Horvath S. (2008). WGCNA: An R Package for Weighted Correlation Network Analysis. BMC Bioinform..

[19] Oksanen J., Blanchet F.G., Kindt R., Legendre P., Minchin P., O’Hara B., Simpson G., Solymos P., Stevens H., Wagner H. (2015). Vegan: Community Ecology Package. R Package, Version 2.2-1.

[20] Ashburner M., Ball C.A., Blake J.A., Botstein D., Butler H., Cherry J.M., Davis A.P., Dolinski K., Dwight S.S., Eppig J.T. (2000). Gene Ontology: Tool for the Unification of Biology. The Gene Ontology Consortium. Nat. Genet..

[21] Aleksander S.A., Balhoff J., Carbon S., Cherry J.M., Drabkin H.J., Ebert D., Feuermann M., Gaudet P., Harris N.L., Gene Ontology Consortium (2023). The Gene Ontology Knowledgebase in 2023. Genetics.

[22] Kanehisa M., Goto S. (2000). KEGG: Kyoto Encyclopedia of Genes and Genomes. Nucleic Acids Res..

[23] Thomas P.D., Ebert D., Muruganujan A., Mushayahama T., Albou L.-P., Mi H. (2022). PANTHER: Making Genome-Scale Phylogenetics Accessible to All. Protein Sci..

[24] Yu G., Wang L.-G., Han Y., He Q.-Y. (2012). clusterProfiler: An R Package for Comparing Biological Themes among Gene Clusters. OMICS.

[25] Ggplot2: Elegant Graphics for Data Analysis (3e). https://ggplot2-book.org/.

[26] Szklarczyk D., Kirsch R., Koutrouli M., Nastou K., Mehryary F., Hachilif R., Gable A.L., Fang T., Doncheva N.T., Pyysalo S. (2023). The STRING Database in 2023: Protein-Protein Association Networks and Functional Enrichment Analyses for Any Sequenced Genome of Interest. Nucleic Acids Res..

[27] Doncheva N.T., Morris J.H., Gorodkin J., Jensen L.J. (2019). Cytoscape StringApp: Network Analysis and Visualization of Proteomics Data. J. Proteome Res..

[28] Chin C.-H., Chen S.-H., Wu H.-H., Ho C.-W., Ko M.-T., Lin C.-Y. (2014). cytoHubba: Identifying Hub Objects and Sub-Networks from Complex Interactome. BMC Syst. Biol..

[29] Evangelista J.E., Xie Z., Marino G.B., Nguyen N., Clarke D.J.B., Ma’ayan A. (2023). Enrichr-KG: Bridging Enrichment Analysis across Multiple Libraries. Nucleic Acids Res..

[30] Xia J., Gill E.E., Hancock R.E.W. (2015). NetworkAnalyst for Statistical, Visual and Network-Based Meta-Analysis of Gene Expression Data. Nat. Protoc..

[31] Yoo M., Shin J., Kim J., Ryall K.A., Lee K., Lee S., Jeon M., Kang J., Tan A.C. (2015). DSigDB: Drug Signatures Database for Gene Set Analysis. Bioinformatics.

[32] Newman A.M., Liu C.L., Green M.R., Gentles A.J., Feng W., Xu Y., Hoang C.D., Diehn M., Alizadeh A.A. (2015). Robust Enumeration of Cell Subsets from Tissue Expression Profiles. Nat. Methods.

[33] Liao M., Liu Y., Yuan J., Wen Y., Xu G., Zhao J., Cheng L., Li J., Wang X., Wang F. (2020). Single-Cell Landscape of Bronchoalveolar Immune Cells in Patients with COVID-19. Nat. Med..

[34] Stuart T., Butler A., Hoffman P., Hafemeister C., Papalexi E., Mauck W.M., Hao Y., Stoeckius M., Smibert P., Satija R. (2019). Comprehensive Integration of Single-Cell Data. Cell.

[35] Gulati G.S., Sikandar S.S., Wesche D.J., Manjunath A., Bharadwaj A., Berger M.J., Ilagan F., Kuo A.H., Hsieh R.W., Cai S. (2020). Single-Cell Transcriptional Diversity Is a Hallmark of Developmental Potential. Science.

[36] Cao J., Spielmann M., Qiu X., Huang X., Ibrahim D.M., Hill A.J., Zhang F., Mundlos S., Christiansen L., Steemers F.J. (2019). The Single-Cell Transcriptional Landscape of Mammalian Organogenesis. Nature.

[37] Jin S., Guerrero-Juarez C.F., Zhang L., Chang I., Ramos R., Kuan C.-H., Myung P., Plikus M.V., Nie Q. (2021). Inference and Analysis of Cell-Cell Communication Using CellChat. Nat. Commun..

[38] R: A Language and Environment for Statistical Computing. https://www.semanticscholar.org/paper/R%3A-A-language-and-environment-for-statistical-Team/659408b243cec55de8d0a3bc51b81173007aa89b.

[39] Pullano G., Di Domenico L., Sabbatini C.E., Valdano E., Turbelin C., Debin M., Guerrisi C., Kengne-Kuetche C., Souty C., Hanslik T. (2021). Underdetection of Cases of COVID-19 in France Threatens Epidemic Control. Nature.

[40] Shin H.-Y. (2021). A Multi-Stage SEIR(D) Model of the COVID-19 Epidemic in Korea. Ann. Med..

[41] Al-Kuraishy H.M., Al-Gareeb A.I., Al-Hussaniy H.A., Al-Harcan N.A.H., Alexiou A., Batiha G.E.-S. (2022). Neutrophil Extracellular Traps (NETs) and Covid-19: A New Frontiers for Therapeutic Modality. Int. Immunopharmacol..

[42] Petrazzuolo A., Le Naour J., Vacchelli E., Gaussem P., Ellouze S., Jourdi G., Solary E., Fontenay M., Smadja D.M., Kroemer G. (2020). No Impact of Cancer and Plague-Relevant FPR1 Polymorphisms on COVID-19. Oncoimmunology.

[43] Kuley R., Duvvuri B., Wallin J.J., Bui N., Adona M.V., O’Connor N.G., Sahi S.K., Stanaway I.B., Wurfel M.M., Morrell E.D. (2023). Mitochondrial N-Formyl Methionine Peptides Contribute to Exaggerated Neutrophil Activation in Patients with COVID-19. Virulence.

[44] Qin S., Yao X., Li W., Wang C., Xu W., Gan Z., Yang Y., Zhong A., Wang B., He Z. (2023). Novel Insight into the Underlying Dysregulation Mechanisms of Immune Cell-to-Cell Communication by Analyzing Multitissue Single-Cell Atlas of Two COVID-19 Patients. Cell Death Dis..

[45] Lee H., Park J., Im H.-J., Na K.J., Choi H. (2021). Discovery of Potential Imaging and Therapeutic Targets for Severe Inflammation in COVID-19 Patients. Sci. Rep..

[46] Li Y., Liu Y., Duo M., Wu R., Jiang T., Li P., Wang Y., Cheng Z. (2022). Bioinformatic Analysis and Preliminary Validation of Potential Therapeutic Targets for COVID-19 Infection in Asthma Patients. Cell Commun. Signal.

[47] Apostolidis S.A., Sarkar A., Giannini H.M., Goel R.R., Mathew D., Suzuki A., Baxter A.E., Greenplate A.R., Alanio C., Abdel-Hakeem M. (2022). Signaling Through FcγRIIA and the C5a-C5aR Pathway Mediate Platelet Hyperactivation in COVID-19. Front. Immunol..

[48] Von Hundelshausen P., Lorenz R., Siess W., Weber C. (2021). Vaccine-Induced Immune Thrombotic Thrombocytopenia (VITT): Targeting Pathomechanisms with Bruton Tyrosine Kinase Inhibitors. Thromb. Haemost..

[49] Zusso M., Lunardi V., Franceschini D., Pagetta A., Lo R., Stifani S., Frigo A.C., Giusti P., Moro S. (2019). Ciprofloxacin and Levofloxacin Attenuate Microglia Inflammatory Response via TLR4/NF-kB Pathway. J. Neuroinflammation.

[50] Frank M.G., Nguyen K.H., Ball J.B., Hopkins S., Kelley T., Baratta M.V., Fleshner M., Maier S.F. (2022). SARS-CoV-2 Spike S1 Subunit Induces Neuroinflammatory, Microglial and Behavioral Sickness Responses: Evidence of PAMP-like Properties. Brain Behav. Immun..

[51] Alves H.R., Lomba G.S.B., Gonçalves-de-Albuquerque C.F., Burth P. (2022). Irisin, Exercise, and COVID-19. Front. Endocrinol..

[52] Liu Z.-M., Yang M.-H., Yu K., Lian Z.-X., Deng S.-L. (2022). Toll-like Receptor (TLRs) Agonists and Antagonists for COVID-19 Treatments. Front. Pharmacol..

[53] Carvalho A., Lu J., Francis J.D., Moore R.E., Haley K.P., Doster R.S., Townsend S.D., Johnson J.G., Damo S.M., Gaddy J.A. (2020). S100A12 in Digestive Diseases and Health: A Scoping Review. Gastroenterol. Res. Pract..

[54] Lei H. (2021). A Single Transcript for the Prognosis of Disease Severity in COVID-19 Patients. Sci. Rep..

[55] Russell C.D., Valanciute A., Gachanja N.N., Stephen J., Penrice-Randal R., Armstrong S.D., Clohisey S., Wang B., Al Qsous W., Wallace W.A. (2022). Tissue Proteomic Analysis Identifies Mechanisms and Stages of Immunopathology in Fatal COVID-19. Am. J. Respir. Cell Mol. Biol..

[56] Arunachalam P.S., Wimmers F., Mok C.K.P., Perera R.A.P.M., Scott M., Hagan T., Sigal N., Feng Y., Bristow L., Tak-Yin Tsang O. (2020). Systems Biological Assessment of Immunity to Mild versus Severe COVID-19 Infection in Humans. Science.

[57] Korbecki J., Maruszewska A., Bosiacki M., Chlubek D., Baranowska-Bosiacka I. (2022). The Potential Importance of CXCL1 in the Physiological State and in Noncancer Diseases of the Cardiovascular System, Respiratory System and Skin. Int. J. Mol. Sci..

[58] Chua R.L., Lukassen S., Trump S., Hennig B.P., Wendisch D., Pott F., Debnath O., Thürmann L., Kurth F., Völker M.T. (2020). COVID-19 Severity Correlates with Airway Epithelium-Immune Cell Interactions Identified by Single-Cell Analysis. Nat. Biotechnol..

[59] Mohamed Y., El-Maradny Y.A., Saleh A.K., Nayl A.A., El-Gendi H., El-Fakharany E.M. (2022). A Comprehensive Insight into Current Control of COVID-19: Immunogenicity, Vaccination, and Treatment. Biomed. Pharmacother..

[60] Şimşek-Yavuz S., Komsuoğlu Çelikyurt F.I. (2021). An Update of Anti-Viral Treatment of COVID-19. Turk. J. Med. Sci..

[61] Naidu S.A.G., Clemens R.A., Pressman P., Zaigham M., Kadkhoda K., Davies K.J.A., Naidu A.S. (2022). COVID-19 during Pregnancy and Postpartum. J. Diet. Suppl..

[62] Masso-Silva J.A., Moshensky A., Lam M.T.Y., Odish M.F., Patel A., Xu L., Hansen E., Trescott S., Nguyen C., Kim R. (2022). Increased Peripheral Blood Neutrophil Activation Phenotypes and Neutrophil Extracellular Trap Formation in Critically Ill Coronavirus Disease 2019 (COVID-19) Patients: A Case Series and Review of the Literature. Clin. Infect. Dis..

[63] Shi S., Nie B., Chen X., Cai Q., Lin C., Zhao G., Zhang X. (2021). Clinical and Laboratory Characteristics of Severe and Non-Severe Patients with COVID-19: A Retrospective Cohort Study in China. J. Clin. Lab. Anal..

[64] Zuo Y., Yalavarthi S., Shi H., Gockman K., Zuo M., Madison J.A., Blair C., Weber A., Barnes B.J., Egeblad M. (2020). Neutrophil Extracellular Traps in COVID-19. JCI Insight.

[65] Chiang C.-C., Korinek M., Cheng W.-J., Hwang T.-L. (2020). Targeting Neutrophils to Treat Acute Respiratory Distress Syndrome in Coronavirus Disease. Front. Pharmacol..

[66] Hazeldine J., Lord J.M. (2021). Neutrophils and COVID-19: Active Participants and Rational Therapeutic Targets. Front. Immunol..

[67] Martinod K., Wagner D.D. (2014). Thrombosis: Tangled up in NETs. Blood.

[68] Torres-Ruiz J., Absalón-Aguilar A., Nuñez-Aguirre M., Pérez-Fragoso A., Carrillo-Vázquez D.A., Maravillas-Montero J.L., Mejía-Domínguez N.R., Llorente L., Alcalá-Carmona B., Lira-Luna J. (2021). Neutrophil Extracellular Traps Contribute to COVID-19 Hyperinflammation and Humoral Autoimmunity. Cells.

[69] Martinod K., Deppermann C. (2021). Immunothrombosis and Thromboinflammation in Host Defense and Disease. Platelets.

[70] Genchi A., Semerano A., Schwarz G., Dell’Acqua B., Gullotta G.S., Sampaolo M., Boeri E., Quattrini A., Sanvito F., Diamanti S. (2022). Neutrophils Predominate the Immune Signature of Cerebral Thrombi in COVID-19 Stroke Patients. Acta Neuropathol. Commun..

