# Supplementary material for: Immune Characteristic Genes and Neutrophil Immune Transformation Studies in Severe COVID-19
Source: Microorganisms. 2024 Apr 4;12(4):737. doi: 10.3390/microorganisms12040737 (PMC11052247; doi:10.3390/microorganisms12040737)
Supplement: Supplementary file 1 [file microorganisms-12-00737-s001.zip › microorganisms-2925187-supplementary/Supplementary File/Figure S1 BAL scRNA-seq cell type definition and disease type distribution.pdf]

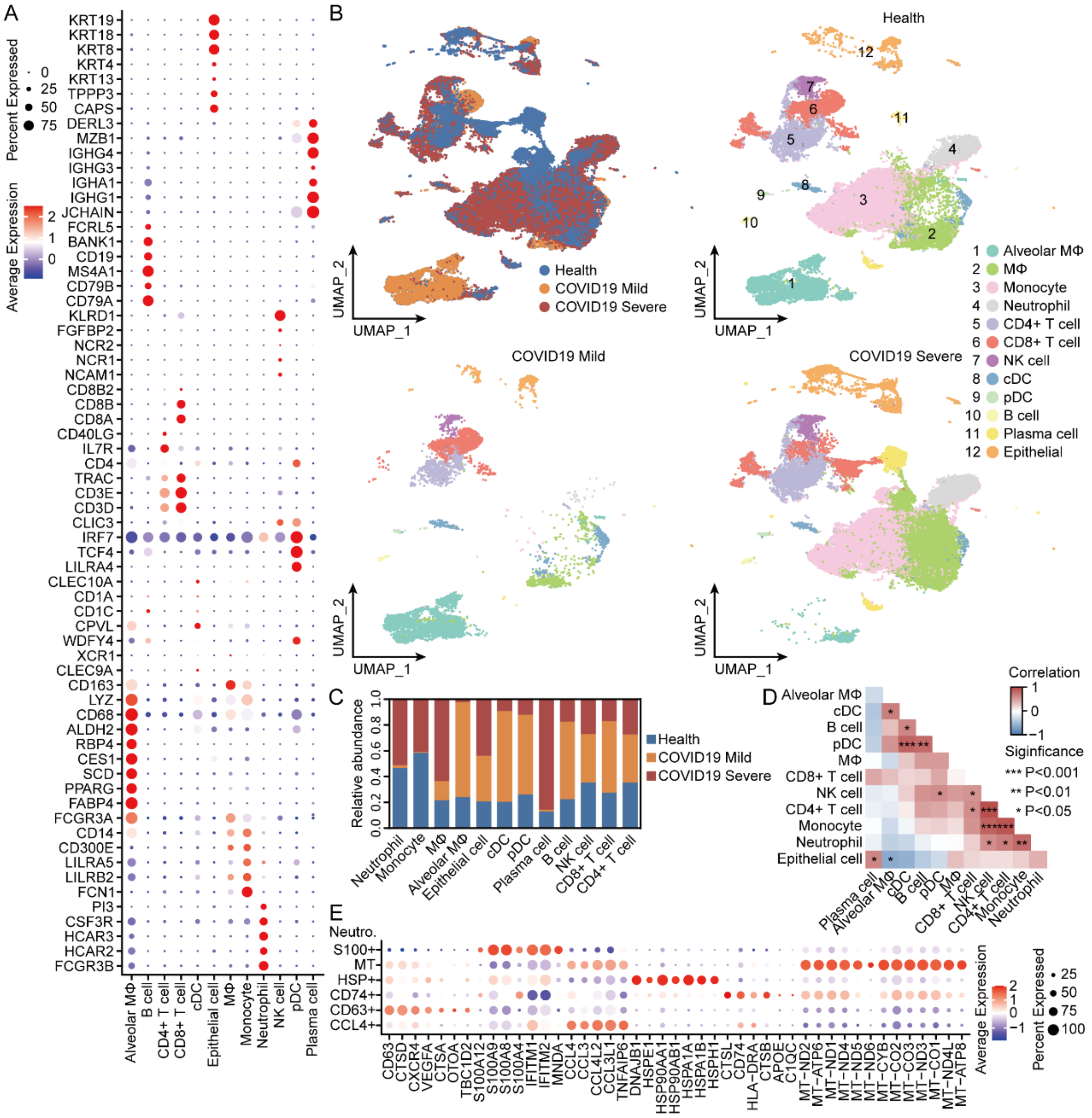

Supplementary Figure S1. BAL scRNA-seq cell type definition and disease type distribution. (A) Marker for major cell type definitions. **(B) and (C)** Distribution and proportion of different major cell types in different disease types. **(D)** Correlation heatmap of major cell types. **(E)** Marker for neutrophil subtype definitions. (* P < 0.05, ** P < 0.01, *** P < 0.001).
